# Modified Clavien-Dindo Classification for Adverse Events in Otolaryngology–Head and Neck Surgery

**DOI:** 10.1001/jamanetworkopen.2025.39761

**Published:** 2025-10-27

**Authors:** Cecelia M. Hidalgo, Eliot J. Martin, Daniel G. Eyassu, Gabriel A. Hernandez-Herrera, Sarah M. Jenkins, Cynthia M. Chweya, George B. Sankar, Semirra L. Bayan, Matthew L. Carlson, Grant S. Hamilton, Eric J. Moore, Joshua P. Wiedermann, Kathryn M. Van Abel

**Affiliations:** 1 Department of Otolaryngology–Head and Neck Surgery, Mayo Clinic, Rochester, Minnesota; 2 Department of Quantitative Health Sciences, Mayo Clinic, Rochester, Minnesota

## Abstract

**Importance:**

Adverse events in otolaryngology–head and neck surgery (OHNS) are inconsistently reported. The Clavien-Dindo Classification has been modified and validated in several surgical subspecialties but has yet to be validated in OHNS.

**Objective:**

To create and validate a modified Clavien-Dindo Classification for OHNS that reflects the severity and duration of harm for complications experienced by patients undergoing OHNS.

**Design, Setting, and Participants:**

This is a multicenter survey study of subject matter experts (SMEs) from OHNS subspecialties from academic institutions within the US. A literature review identified publications reporting surgical complications in OHNS in the English language between January 2019 and April 2020. Complications were summarized and used to inform a modified Clavien-Dindo Classification. Feedback from internal SMEs was incorporated. Thirty-five clinical vignettes were created, and an electronic survey was sent to SMEs at outside institutions. Each SME was randomly assigned to 1 of 4 groups to evaluate 8 to 9 vignettes. The survey was conducted in September 2023, and data were analyzed from February to May 2024.

**Exposure:**

Participation in the electronic survey.

**Main Outcomes and Measures:**

Vignette scores were assessed and the Comprehensive Complication Index (CCI) calculator was used to assess cumulative morbidity. The scale is based on the grading by the Clavien-Dindo Classification and reflects overall morbidity on a scale from 0 (no complication) to 100 (death). Variability of the CCIs between reviewers, the primary outcome, was summarized with the SD. The percentage of reviewers with a high (≥26.2) or low (<26.2) CCI within a vignette was reported. Reliability was summarized using Krippendorff α.

**Results:**

Of 106 SMEs, 43 (response rate, 40.6%) completed at least 1 vignette. Eighteen of 35 vignettes (51.4%; 95% CI, 34.9%-68.0%) had unanimous high CCI, and 8 of 35 vignettes (22.9%; 95% CI, 8.9%-36.8%) had unanimous low CCI (overall categorical agreement, 74.3%; 95% CI, 59.8%-88.8%). On average, the Krippendorff α was 0.74, indicating moderate reliability. A total of 21 850 OHNS complications were reviewed and categorized as surgical or medical with 12 including subcategorization for specificity. Each category was then assigned a modified OHNS Clavien Dindo classification grade.

**Conclusions and Relevance:**

In this survey study of OHNS experts, a modified OHNS Clavien-Dindo Classification was developed and validated using case vignettes. The modified Clavien-Dindo Classification provided a granular subcategorization for each complication category and grade, duration of harm, and an overall summary complication score (CCI). Results suggest the modified OHNS Clavien-Dindo Classification reliably differentiated between high-grade and low-grade OHNS complications and could be utilized for assessing surgical outcomes.

## Key Points

**Question:**

Can the Clavien-Dindo Classification be modified and validated for otolaryngology–head and neck surgery (OHNS)?

**Findings:**

In this survey study, a modified Clavien-Dindo Classification for surgical complications in OHNS was created and validated by OHNS experts using case vignettes, resulting in moderate agreement among 106 survey responders.

**Meaning:**

There are unique surgical complications encountered in OHNS that could be captured using a novel modified Clavien-Dindo Classification, which was reliable in differentiating between severity of OHNS complications.

## Teaser

This survey study examines a modified Clavien-Dindo Classification system for adverse events in otolaryngology–head and neck surgery.

## Introduction

In 1992, Clavien et al^1^ proposed a system to classify complications according to the interventions required to correct them, addressing the need for a standardized surgical complication reporting system. In 2004, Dindo et al^2^ revised this scale and since then, the Clavien-Dindo Classification has been cited extensively, providing a widely accepted and reproducible reporting system for surgical complications.^3^ The Clavien-Dindo Classification has been modified and validated within subspecialities, such as orthopedic, cardiovascular, and urologic surgery,^4,5,6^ to better characterize and grade procedure-specific complications. For instance, Hébert et al^5^ included the use of antihypertensives, antiarrhythmics, vasopressors, and vasodilators within the grade I definition given their routine use within cardiac surgery. However, to date, a similar validation has not been performed for otolaryngology–head and neck surgery (OHNS).

Although it is valuable, the Clavien-Dindo Classification has important limitations when applied to OHNS. It fails to capture complications routinely encountered within the specialty, such as injury to motor and sensory nerves of the head and neck, which may limit its usefulness for investigators when assessing a specific surgical procedure.^7^ Some modifications have been proposed; for instance, Jan et al^8^ incorporated “partial or total free flap failure” into the grade III definition to better capture complications of free flap reconstruction. Despite these efforts, adverse event (AE) reporting within OHNS publications remains inconsistent. For example, complications arising from transoral robotic surgery are heterogeneously reported in the literature. Chia et al^9^ categorized transoral robotic surgery complications using descriptors such as *minor* and *major*, whereas the ECOG3311 and ORATOR clinical trials graded oropharyngeal bleeding according to the Common Terminology Criteria for Adverse Events.^10,11^ Similar disconnects exist in reporting thyroid surgery complications, where hypocalcemia may or may not be symptomatic, with additional temporal descriptors like *transient* or *permanent* failing to capture the full spectrum of this AE.^12,13,14^

Moreover, the Clavien-Dindo Classification does not indicate the duration of harm or provide an overall AE score. To address this, the Comprehensive Complication Index (CCI) was developed to assess the total number of complications in a single patient, allowing comparisons across patients and studies.^15,16^ In addition, validated tools such as the Agency for Healthcare Research and Quality (AHRQ) Common Format Harm Scale use the duration of harm—temporary (<12 months), permanent (>12 months), or unknown—to define each AE.^17^ However, neither tool was designed for surgical complication reporting in OHNS. The CCI summarizes cumulative burden but does not specify the nature or duration of harm, whereas the AHRQ scale captures duration but lacks gradation by intervention. Combining the utility of the Clavien-Dindo Classification with the CCI’s cumulative score and the AHRQ’s duration framework, investigators can achieve a standardized reproducible tool for assessing surgical outcomes. Thus, this study aimed to create and validate a modified Clavien-Dindo Classification for OHNS that reflects unique AEs associated with surgery, severity, and duration of harm.

## Methods

### Study Design

This survey study aimed to validate a modified Clavien-Dindo Classification adapted for OHNS by conducting a literature review for reported OHNS complications, modifying the Clavien-Dindo Classification on the basis of those complications, and validating the modified system through a survey of subject matter experts (SMEs). Approval for this study was obtained from the Mayo Clinic institutional review board. This report followed the American Association for Public Opinion Research (AAPOR) reporting guideline for survey studies. Survey development, electronic distribution, and reporting were performed in collaboration with the Mayo Clinic Survey Research Center, which adheres to AAPOR standards for survey design, participant recruitment, and response documentation. Participants’ completion and submission of the survey served as consent to participant in the study.

### Literature Review

A literature review identified publications that reported surgical complications in OHNS in the English language between January 2019 and April 2020. Complications identified during the literature review were summarized, quantified, and categorized into surgical or medical complications. We then used these summarized data to inform a modified Clavien-Dindo Classification for OHNS.

### Modification of the Original Clavien-Dindo Classification

The original Clavien-Dindo Classification stratifies surgical complications into 5 grades: grade I (any deviation from the normal postoperative course without the need for pharmacological treatment), grade II (requiring pharmacological treatment, blood transfusion, or total parental nutrition), grade III (requiring surgical, endoscopic, or radiological intervention, with IIIa not under general anesthesia and IIIb under general anesthesia), grade IV (life-threatening complication requiring intensive care unit management, with IVa representing single-organ dysfunction and IVb multiorgan dysfunction), and grade V (death).

In the modified Clavien-Dindo Classification, grades IV and V remained the same but grades I to III were adapted to better capture the spectrum of OHNS complications. Grade I was redefined as a low-risk deviation requiring conservative measures, grade II as a high-risk deviation requiring minimal intervention, and grade III as complications necessitating intensive care unit care or surgical or procedural intervention, with IIIa denoting minimally invasive procedures and IIIb denoting invasive, urgent, or emergent procedural interventions. The modified system further classified complications by the duration of harm, differentiating them as temporary (<12 months), permanent (>12 months), or unknown. Finally, SMEs from each OHNS subspecialty (head and neck, laryngology, otology-neurotology, rhinology-skull base, pediatrics, and facial plastic and reconstructive surgery) at our institution performed a formal review of the modified system. Collective feedback was used to further revise and finalize the grading rubric (eTable 1 in Supplement 1) and modified grading scale.

### Face Validation Surveys

Participants were recruited through our institution’s OHNS SMEs, who each provided email addresses for 10 practicing otolaryngologists in their respective subspecialties. We developed 35 hypothetical clinical vignettes (see the eAppendix in Supplement 1 for detailed clinical vignettes) reflecting complications within the modified Clavien-Dindo Classification. Working with the Mayo Clinic Survey Research Center, an electronic survey was distributed through email to the SMEs. Each SME was randomly assigned to 1 of 4 groups to evaluate 8 to 9 vignettes. For each vignette, SMEs were asked to select all surgical complications within 30 days of surgery by selecting from the list of surgical and medical complication categories. Because of the complexity of the survey, each vignette was sent in a separate email to the reviewer. For each complication, the SMEs were asked to identify the intervention the patient received and assess the duration of harm from the complication. The CCI calculator was used to assess the cumulative morbidity following the intervention presented in the vignette. The scale is based on the complication grading by the Clavien-Dindo Classification and reflects overall morbidity on a scale from 0 (no complication) to 100 (death).^16,18^ The survey was conducted in September 2023, and data were analyzed from February to May 2024.

### Statistical Analysis

The 35 vignettes were randomized across 4 surveys forms, with a different set of reviewers for each survey group. Within each vignette, the variability of the CCIs between the reviewers was summarized with the SD and was classified as high (SD ≥10), moderate (SD 3 to <10), or low (SD <3), according to the observed distribution in the data. We utilized a CCI of 26.2, equivalent to 1 Clavien-Dindo Classification grade IIIa complication, as the cutoff value between high (≥26.2) and low (<26.2) CCI, consistent with the literature.^19,20,21,22^ The percentage of reviewers with a high or low CCI within a vignette was reported, and categorical agreement was defined as vignettes in which all reviewers provided a categorically equivalent score. Percentages were summarized along with 95% CIs as appropriate. Across vignettes within each survey, interrater reliability was summarized using Krippendorff α^23^ treating the CCIs continuously. Krippendorff α can range from −1 to 1, with a value of 0 indicating no reliability, a value of 1 indicating high reliability, and a value of −1 indicating low reliability. We regarded Krippendorff α 0.80 or higher as satisfactory reliability, 0.67 to 0.79 as moderate, and less than 0.67 as poor.^24^ Analyses were performed using R statistical software version 4.2.2 (R Project for Statistical Computing),^25^ including the kripp.alpha function within the irr package.^26^

## Results

Nine hundred thirty-seven studies were identified, and, ultimately, 222 studies met inclusion criteria. A total of 21 850 complications were reported across 50 928 patients (eTable 2 in Supplement 1). Of the articles reviewed, 18 (8.1%; 95% CI, 4.5%-11.7%) utilized the Clavien-Dindo Classification in their reporting. These complications were then categorized as surgical or medical, with 12 including subcategorization for specificity. Each category was then assigned a modified OHNS Clavien-Dindo Classification grade.

Five internal SMEs provided comprehensive feedback and once a final version reached consensus, the survey was converted to an electronic format and external SMEs were contacted for validation of the tool. Among 106 SMEs contacted to participate in the survey, 43 completed assessment of at least 1 vignette (response rate, 40.6%). The 3 most common subspecialties among the respondents were head and neck (11 respondents [25.6%]), facial plastic and reconstructive surgery (9 respondents [20.9%]), and laryngology (6 respondents [14.0%]) (Table 1).

**Table 1.** Sample Characteristics

Characteristic	Subject matter experts, No. (%) (N = 106)
Institution	
Index	41 (38.7)
Outside	65 (61.3)
Response rate, No. of responders/total No. (%)^a^	
Index institution	21/41 (51.2)
Outside institution	22/65 (33.8)
Subspecialty	
Facial plastic and reconstructive surgery	9 (20.9)
General otolaryngology–head and neck surgery	3 (7.0)
Head and neck	11 (25.6)
Laryngology	6 (14.0)
Otology	5 (11.6)
Pediatrics	4 (9.3)
Rhinology	5 (11.6)

^a^ In total, 43 individuals completed at least 1 vignette, for a response rate of 40.6%. Among the 63 nonresponders, 5 opted out, and 58 provided no response.

The variability for each vignette is summarized in Table 2 and illustrated in the Figure. Even in the presence of moderate to high variability, there were 18 of the 35 vignettes (51.4%; 95% CI, 34.9%-68.0%) in which all reviewer responses aligned with a high CCI, and 8 vignettes (22.9%; 95% CI, 8.9%-36.8%) in which all reviewer responses aligned with a low CCI (overall categorical agreement in 74.3% of scenarios; 95% CI, 59.8%-88.8%). On average, the Krippendorff α was 0.74 (moderate reliability), with α values of 0.69 (moderate) for survey group 1, 0.86 (satisfactory) for survey group 2, 0.83 (satisfactory) for survey group 3, and 0.58 (poor) for survey group 4 (Figure).

**Table 2.** Summary of Clinical Vignettes

Survey group and vignette No.	No. of reviewers	High CCI, No. (%)^a^	Low CCI, No. (%)^a^	CCI, mean (SD)^b^	Variability^c^
Group 1					
1	16	1 (6)	15 (94)	13.2 (6.9)	Moderate
2	15	0	15 (100)	18.5 (5.1)	Moderate
3	14	14 (100)	0	30 (4.5)	Moderate
4	14	14 (100)	0	30.5 (7.0)	Moderate
5	14	14 (100)	0	100 (0)	Low
6	12	2 (17)	10 (83)	10.2 (8.2)	Moderate
7	14	14 (100)	0	28.5 (5.9)	Moderate
8	14	14 (100)	0	42.7 (7.0)	Moderate
Group 2					
9	9	9 (100)	0	47.9 (12.3)	High
10	6	0	6 (100)	8.7 (0)	Low
11	4	0	4 (100)	8.7 (0)	Low
12	6	6 (100)	0	38.2 (6.9)	Moderate
13	5	0	5 (100)	21.2 (0.8)	Low
14	5	0	5 (100)	20.9 (0)	Low
15	5	5 (100)	0	27.7 (3.4)	Moderate
16	5	3 (60)	2 (40)	21.6 (7.6)	Moderate
17	5	5 (100)	0	40.7 (3.9)	Moderate
Group 3					
18	10	10 (100)	0	57.6 (31.6)	High
19	9	1 (11)	8 (89)	21.9 (2.9)	Low
20	9	1 (11)	8 (89)	21.9 (2.9)	Low
21	9	9 (100)	0	44.3 (11.2)	High
22	9	9 (100)	0	42.5 (6.8)	Moderate
23	9	0	9 (100)	8.7 (0)	Low
24	9	0	9 (100)	8.7 (0)	Low
25	9	9 (100)	0	48 (4.6)	Moderate
26	9	6 (67)	3 (33)	31.6 (10.7)	High
Group 4					
27	8	5 (63)	3 (38)	28.9 (21.5)	High
28	4	4 (100)	0	39.7 (0)	Low
29	6	6 (100)	0	81.5 (16)	High
30	6	5 (83)	1 (17)	29.9 (10.4)	High
31	6	3 (50)	3 (50)	28.9 (14.2)	High
32	6	6 (100)	0	100 (0)	Low
33	6	6 (100)	0	34.0 (4.5)	Moderate
34	6	6 (100)	0	54.2 (11.2)	High
35	6	0	6 (100)	9.3 (1.4)	Low

Abbreviation: CCI, Comprehensive Complication Index.

^a^ Refers to the percentage of reviewers within a vignette with a high vs a low CCI, calculated from their responses. A high CCI was defined as 26.2 or higher, whereas a low CCI was defined as less than 26.2.

^b^ Refers to SDs for the CCIs from the set of reviewers within a vignette.

^c^ The variability of reviewer CCIs within a vignette was categorized as high (SD ≥10), moderate (SD 3 to <10), or low (SD <3).

**Figure.**
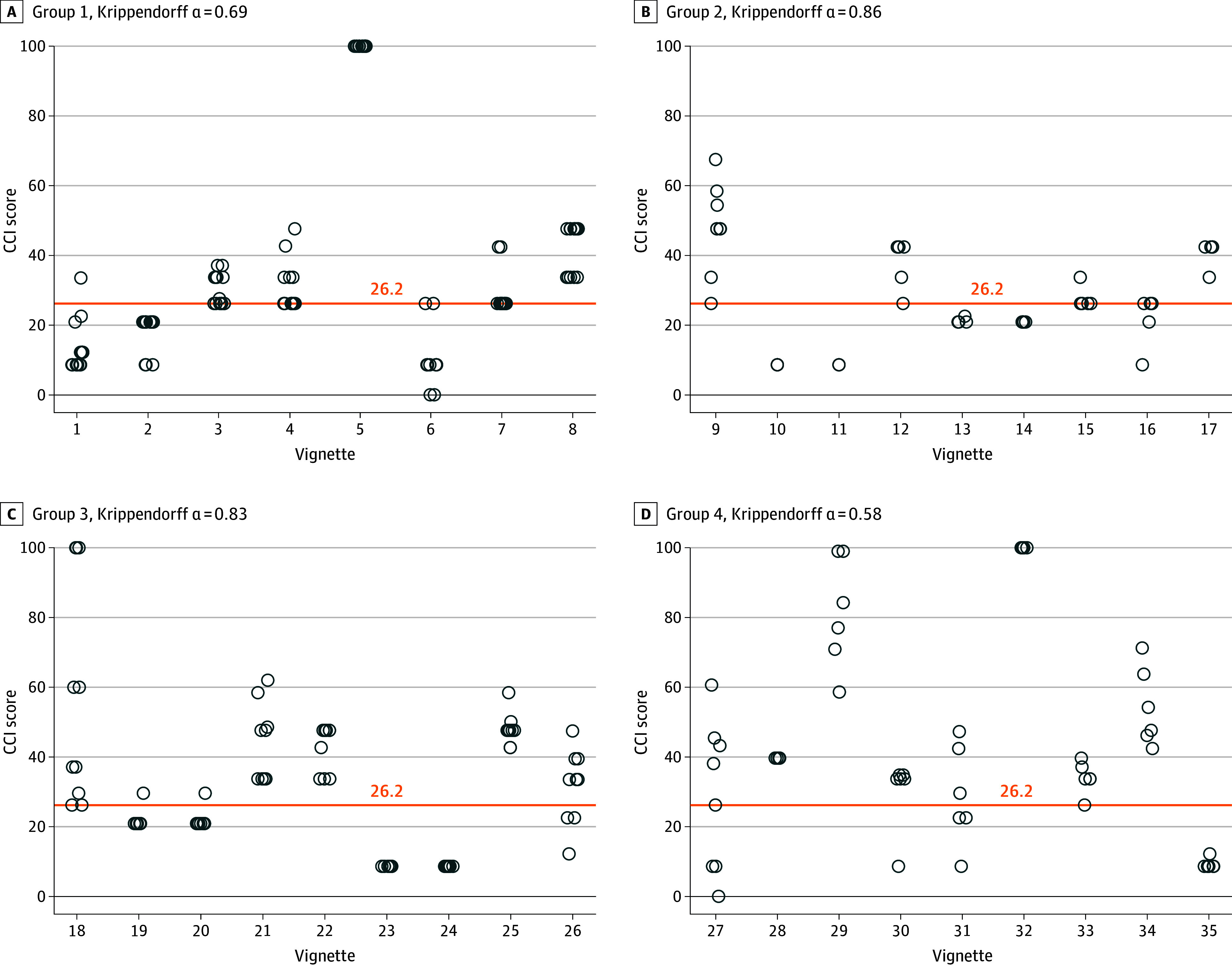
Comprehensive Complication Index (CCI) and Krippendorff α Krippendorff α values 0.80 or higher denote satisfactory reliability, values of 0.67 to 0.79 denote moderate reliability, and values less than 0.67 denote poor reliability.

Of the vignettes for which all SMEs provided a high CCI with low variability (vignettes 5, 28, and 32), the complications were death, urinary retention, and hemorrhage. Of the vignettes for which all SMEs graded a low CCI with low variability (vignettes 10, 11, 13, 14, 23, 24, and 35), the complications were postoperative fluid or air collection (sialocele), motor nerve injury, surgical site infection, device or implant concern, pain management, chyle leak, and sensory or autonomic nerve injury. Of the vignettes for which SMEs did not unanimously grade the complication with a high or low CCI and there was high variability (vignettes 26, 27, 30, and 31), the complications were surgical site infection, nerve injury (motor, sensory or autonomic), pain management, device or implant concern, dysphagia, and nausea or vomiting. Much of the disagreement stemmed from some reviewers assuming a complication was surgical, whereas others categorized the same complication as medical. Twenty-six of 35 vignettes (74.3%; 95% CI, 59.8%-88.8%) had complete agreement on duration of harm, and the 9 vignettes in which there was disagreement are detailed in eTable 3 in Supplement 1.

## Discussion

In this survey study, we created and validated a modified Clavien-Dindo Classification for OHNS, refining it to reflect complications commonly reported across OHNS procedures while downgrading assessments like nasopharyngolaryngoscopy, which is routinely used to assess postoperative patients. Our results suggest that the modified OHNS Clavien-Dindo Classification is reliable in differentiating between high and low-grade OHNS complications.

The modified scale’s performance varied on the basis of the complexity of the vignette presented to the SME. Vignettes in which the tool performed well (100% agreement on dichotomous CCI outcome and low variability) typically contained a single complication with the outcome and intervention clearly stated. For example, explicit wording in the clinical vignette, such as “Abscess is opened in clinic revealing 10 cc of purulent material” and “Patient is swiftly taken to the operating room,” allowed SMEs to correctly choose between “Wound exploration ± packing in non-operative setting” (grade II complication) and “Urgent or emergent surgical procedure” (grade IIIb complication), respectively. Vignettes in which the tool performed poorly generally contained more than 1 complication, such as a patient presenting with postoperative intractable nausea and vomiting as well as leftward tongue deviation and dysarthria. The format of assessment education and instructions when using this tool may be critical in successfully grading each complication. The ability to view instructions, the full vignette, and more than a single selected complication at a time was limited using the online survey platform, which likely negatively impacted consistent grading, especially for multiple complications. In addition, the need to complete a vignette, exit the application, return to a SMEs’ email, and open the next vignette likely negatively impacted the completion of all vignettes. Future analysis should allow reviewers to view the grading rubric on a single document when assessing each clinical vignette.

Many OHNS patients experience both medical and surgical complications in the perioperative period. It is critical to be able to assess each complication and its overall impact on the patient. This allows more complete comparison across treatment modalities, such as surgery vs radiosurgery for vestibular schwannoma^27^ and operative vs nonoperative therapy for upper aerodigestive tract tumors.^11^ In our assessment, it was challenging for SMEs to differentiate when a complication should be considered surgical or medical. For example, in vignettes with a complication of surgical site infection, some SMEs selected interventions under both surgical (surgical site infection) and medical (infectious) categories, resulting in a higher CCI. There was also disagreement among SMEs regarding the definition of organ failure. Some viewed sensory nerve injury (hearing loss) and motor nerve injury (tongue deviation) as a single organ failure but others did not, resulting in widely dispersed CCIs (eg, vignette 31 in the Figure). Further optimization of the tool to provide education and instructions to investigators utilizing this tool will be critical.

Patients undergoing OHNS often experience complications that occur within 30 days of surgery, but treatment for the complication is delayed until after completion of the initial treatment. For patients, this treatment is experienced as related to the initial complication. It is critical that we are able to assess the overall burden of our treatments for our patients.^28^ In our study, timing of the intervention was another cause of varying CCIs as some SMEs only included interventions that occurred within the postoperative 30-day period. For instance, in vignette 26, a brow lift was scheduled at a 1-year postoperative appointment for a complete ipsilateral brow paralysis that occurred on postoperative day 1 after an elective rhytidectomy. Our scale is intended to include any surgical intervention for a complication that occurred within 30 days following surgery, so in this vignette, a planned surgical procedure in an operative setting (grade IIIa) should be indicated as an intervention for cranial nerve VII injury. This offers key insight into AE reporting and creates an opportunity for education and instructions to ensure complete and consistent reporting of patient experienced such surgical complications.

Sink et al^4^ adapted the Clavien-Dindo Classification for orthopedic surgery and tested its reliability for hip preservation surgery. Several aspects of their methods may have contributed to their excellent interobserver and intraobserver reliability. The authors trained their readers in the classification system at an in-person meeting and provided definitions for the orthopedic equivalents of organ dysfunction, and readers had access to the classification system while grading scenarios. A training session describing the modified OHNS Clavien-Dindo Classification and providing OHNS equivalents for organ dysfunction may mitigate some of the disagreement among SMEs in our study. As stated previously, the survey user experience likely limited interobserver agreement; the ability to visualize the full OHNS Clavien-Dindo Classification rubric while grading will be important for further validation. Our modified grading scale is characterized by a high level of detail, which leads to increased complexity and length but improved granularity in reporting outcomes of interest to OHNS SMEs. Although the Common Terminology Criteria for Adverse Events, the most commonly used AE grading tool among radiation and medical oncologists, contains 26 different categories and is more than 140 pages long,^29^ it lacks specificity for surgical complication assessment.

A notable strength of the modified Clavien-Dindo Classification is its granularity, which offers a detailed framework for precise categorization of complications. The tool has tailored subcategories for complications, such as those for cranial nerve injuries, that reflect the challenges specifically encountered in the specialty as well as their anticipated interventions. This allows for capturing and appropriately grading complications that may otherwise be inadequately reported or described. For example, although taste disturbance is a complication that impacts patient quality of life, it has been described to be underreported or vaguely described in the literature.^30^ Our modified Clavien-Dindo Classification effectively captures and categorizes the severity of altered taste and tongue numbness following middle ear surgery for cholesteatoma, as illustrated by the unanimous low CCI scores and low variability among SMEs who graded vignette 35.

In addition, incorporating the CCI into our grading system improves the global assessment of a patient’s experience by considering complications of all grades that the patient endures rather than focusing on the single most severe complication experienced. There was overall CCI categorical agreement in 74.3% of our scenarios, illustrating that the cumulative burden of all postoperative complications is accurately captured, thus allowing researchers and clinicians to better assess the severity and impact of complications on a patient’s experience. With increasing clinician use, clear instructions on use, and resultant familiarity with our modified Clavien-Dindo Classification, we anticipate substantial elevation of categorical agreement with future study application.

Finally, including duration of harm in our grading scale provides an added dimension for evaluating and documenting long-term outcomes. Williams et al^17^ found that both versions of the AHRQ Common Format Harm Scale had moderate interrater reliability, supporting the use of the scale in clinical settings. Dindo et al^2^ included the suffix “d” for disability to indicate complications that have the potential for long-term disability after discharge, but a recent study by Abbassi et al^16^ found that a minority of respondents find this suffix useful, and it was rarely utilized in randomized clinical trials. For these reasons, our scale utilizes a cutoff point of 12 months to categorize duration of harm. There was 100% agreement of duration of harm in 26 of the 35 clinical vignettes (74.3%), illustrating that most SMEs agree on the long-term impact a complication has on a patient. The specificity and versatility of our tool reduces ambiguity and improves consistency in reporting OHNS complications. This allows for generating reproducible data for cross-institutional comparisons and potentially for clinical benchmarking for surgical quality and outcomes monitoring.

### Limitations

Given the nature of survey research, our study is limited by nonresponse bias. We believe our response rate of 40.6% is quite robust given that the survey sample consisted of OHNS attending surgeons who voluntarily participated in the survey without authorship recognition. This response rate is similar to a previously published study that surveyed head and neck surgeons.^7^ The complexity and length of our survey, which included 8 to 9 detailed clinical vignettes distributed in 8 to 9 separate emails, may have contributed to a lower completion rate and likely affected the consistency of the responses. This study did not collect formal feedback from external SMEs regarding the ease and feasibility of applying the modified Clavien-Dindo Classification. Future studies should evaluate usability to ensure the scale can be implemented effectively in clinical and research settings. Finally, the inability to see the entirety of the modified Clavien-Dindo Classification during grading after a complication was selected likely limited accuracy of grading. In the future, viewing the entire modified OHNS Clavien-Dindo Classification on a single page and improved instructions clarifying medical vs surgical complications and how to manage multiple complications will be critical. Finally, it may be useful for certain subspecialities to abridge the grading scale to only include surgical and medical complications that are typically relevant to their patient population and surgical techniques.

## Conclusions

By providing a granular subcategorization for each complication category and grade, a duration of harm, and a summary complication score (CCI), our modified classification addresses several limitations of using the Clavien-Dindo Classification to grade surgical complications in OHNS. The complexity of our grading rubric may limit its utility in the clinical setting but will allow for more nuanced analysis of surgical outcomes reporting in research. Further study is needed to assess its performance in comparison to other available tools.

## Notes

Supplement 1. eTable 1. Grading Rubric for Oto—HNS Modified CDC eAppendix. Supplemental Clinical Vignettes eTable 2. Literature Review Oto—HNS Complication Frequency Summary eTable 3. Duration of Harm: Distribution of SME Responses

Supplement 2. Data Sharing Statement
