# Error in Table 2

**DOI:** 10.1001/jamanetworkopen.2025.50091

**Published:** 2025-11-26

In the Original Investigation titled “Modified Clavien-Dindo Classification for Adverse Events in Otolaryngology–Head and Neck Surgery,”^1^ published October 27, 2025, there was an error in Table 2. In Table 2, the column reflecting the mean (SD) Comprehensive Complication Index (CCI) score was labeled as SD but should have been labeled: CCI, mean (SD). This article has been corrected.^1^
