# Supplementary material for: Modified Clavien-Dindo Classification for Adverse Events in Otolaryngology–Head and Neck Surgery
Source: JAMA Netw Open. 2025 Oct 27;8(10):e2539761. doi: 10.1001/jamanetworkopen.2025.39761 (PMC12559966; doi:10.1001/jamanetworkopen.2025.39761)
Supplement: Supplement 1. — eTable 1. Grading Rubric for Oto—HNS Modified CDC eAppendix. Supplemental Clinical Vignettes eTable 2. Literature Review Oto—HNS Complication Frequency Summary eTable 3. Duration of Harm: Distribution of SME Responses [file jamanetwopen-e2539761-s001.pdf]

Supplemental Online Content

Hidalgo CM, Martin EJ, Eyassu DG, et al. Modified Clavien-Dindo classification for adverse events in otolaryngology–head and neck surgery. *JAMA Netw Open*. 2025;8(10):e2539761. doi:10.1001/jamanetworkopen.2025.39761

eTable 1. Grading Rubric for Oto—HNS Modified CDC

eAppendix. Supplemental Clinical Vignettes

eTable 2. Literature Review Oto—HNS Complication Frequency Summary

eTable 3. Duration of Harm: Distribution of SME Responses

This supplemental material has been provided by the authors to give readers additional information about their work.

© 2025 Hidalgo CM et al. *JAMA Network Open*.

eTable 1. Grading Rubric for Oto—HNS Modified CDC

Complication related to surgery diagnosed within 30 days of surgery[a]	Grade I<br>Low risk deviation from the normal post op course	Grade II<br>High risk deviation from the normal post op course.	Grade IIIa<br>Low to moderately invasive surgery or procedure or intensive care unit (ICU) care.	Grade IIIb<br>Invasive, urgent or emergent surgical intervention or procedure.	Grade IVa<br>Severe complication – e.g., single organ failure.	Grade IVb<br>Life threatening complication – e.g., multi organ failure.	Grade V<br>Death	Duration of Harm
Normal post-operative course	Allowed: drugs such as antiemetics, antipyretics, analgesics, diuretics, electrolytes, planned dietary supplementation and physiotherapy.							
**Surgical**								
Airway control<br>☐ Hypoxic brain injury	☐ Supplemental oxygen<br>☐ Oral medications<br>☐ Inhaled medications<br>☐ Nebulized medications<br>☐ Unplanned endoscopic exam<br>☐ Concern resulting in Emergency room evaluation	☐ IV medications<br>☐ Wound exploration ± packing in non-operative setting<br>☐ Non-invasive positive pressure<br>☐ Concern resulting in hospital admission	☐ Unexpected intubation<br>☐ Unplanned mechanical ventilation <24 hours<br>☐ Planned surgical procedure in an operative setting<br>☐ Planned interventional radiology procedure<br>☐ Concern resulting in ICU admission	☐ Urgent or emergent surgical procedure<br>☐ Unplanned mechanical ventilation > 24 hours	☐ Single organ failure secondary to loss of airway	☐ Multi organ failure secondary to loss of airway	☐ Death attributed to loss of airway	☐ Temporary (< 12 months)<br>☐ Permanent (> 12 months)<br>☐ Unknown
Pain Management	☐ Local analgesics<br>☐ Oral analgesics<br>☐ Concern resulting in Emergency room evaluation	☐ IV analgesics, bolus<br>☐ Wound exploration in non-operative setting	☐ IV analgesics, patient-controlled pump<br>☐ IV sedation, *not* general anesthesia	☐ Urgent or emergent surgical procedure<br>☐ Unplanned mechanical ventilation > 24 hours	☐ Single organ failure secondary to analgesia or sedation	☐ Multi organ failure secondary to analgesia or sedation	☐ Death attributed to analgesia or sedation	☐ Temporary (< 12 months)<br>☐ Permanent (> 12 months)<br>☐ Unknown

© 2025 Hidalgo CM et al. *JAMA Network Open*.

		☐ Oral sedative<br>☐ Nerve block<br>☐ Concern resulting in hospital admission	☐ Nerve block or pump requiring sedation<br>☐ Planned surgical procedure in an operative setting<br>☐ Planned interventional radiology procedure<br>☐ Unplanned mechanical ventilation for < 24 hours<br>☐ Concern resulting in ICU admission						
Chyle leak	☐ NPO ≤ 72 hours<br>☐ Dietary modification<br>☐ Needle aspiration<br>☐ Compression wrap<br>☐ Oral medications<br>☐ Intramuscular and/or subcutaneous injection<br>☐ Prolonged drain requirement<br>☐ Concern resulting in Emergency room evaluation	☐ NPO > 72 hours<br>☐ Electrolyte management<br>☐ Wound exploration ± packing in non-operative setting<br>☐ IV medications<br>☐ Concern resulting in hospital admission	☐ Total parenteral nutrition (TPN)<br>☐ Planned surgical procedure in an operative setting<br>☐ Planned interventional radiology procedure<br>☐ Concern resulting in ICU admission	☐ Urgent or emergent surgical procedure	☐ Single organ failure secondary to chyle leak or sequelae	☐ Multi organ failure secondary to chyle leak or sequelae	☐ Death attributed to chyle leak or sequelae	☐ Temporary (< 12 months)<br>☐ Permanent (> 12 months)<br>☐ Unknown

© 2025 Hidalgo CM et al. *JAMA Network Open*.

Cerebrospinal fluid (CSF) leak<br>☐ Anterior skull base CSF leak<br>☐ Lateral skull base CSF leak<br>☐ Tension pneumocephalus<br>☐ CSF leak complicated by intracranial infection<br>☐ Brain herniation	☐ Bed rest or related conservative measures<br>☐ Compression wrap<br>☐ Oral medications<br>☐ Concern resulting in Emergency room evaluation	☐ IV medications<br>☐ Lumbar drain placement<br>☐ Wound exploration ± packing in non-operative setting<br>☐ Concern resulting in hospital admission	☐ Extraventricular drain placement<br>☐ Planned surgical procedure in an operative setting<br>☐ Planned interventional radiology procedure<br>☐ Concern resulting in ICU admission	☐ Urgent or emergent surgical procedure	☐ Single organ failure secondary to CSF leak or sequelae	☐ Multi organ failure secondary to CSF leak or sequelae	☐ Death attributed to CSF leak	☐ Temporary (< 12 months)<br>☐ Permanent (> 12 months)<br>☐ Unknown
Death							☐ Unknown cause<br>☐ Other, please specify:	☐ Permanent (> 12 months)
Dysphagia	☐ Modified diet, no *unexpected* supplemental enteral nutrition<br>☐ Concern resulting in Emergency room evaluation<br>☐ Unplanned endoscopic exam	☐ Delayed nasogastric tube placement<br>☐ IV fluid resuscitation<br>☐ Refeeding syndrome<br>☐ Aspiration pneumonia not requiring ICU admission<br>☐ Concern resulting in hospital admission	☐ Planned surgical procedure in an operative setting<br>☐ Planned interventional radiology procedure<br>☐ PEG, G or J-tube placement<br>☐ Total parenteral nutrition (TPN)<br>☐ Unintentional weight loss ≤ 10%	☐ Unintentional weight loss > 10%<br>☐ Urgent or emergent surgical procedure	☐ Single organ failure secondary to dysphagia or sequelae	☐ Multi-organ failure secondary to dysphagia or sequelae	☐ Death attributed to dysphagia or sequelae	☐ Temporary (< 12 months)<br>☐ Permanent (> 12 months)<br>☐ Unknown

© 2025 Hidalgo CM et al. *JAMA Network Open*.

			☐ Concern resulting in ICU admission					
Endocrine dysfunction<br><br>☐ Hypocalcemia<br>☐ Hypothyroidism<br>☐ Hypoparathyroidism<br>☐ Diabetes insipidus<br>☐ Other, please specify:	☐ Oral medications<br>☐ Modified diet or fluid intake<br>☐ Concern resulting in Emergency room evaluation	☐ IV medications<br>☐ Concern resulting in hospital admission	☐ Planned surgical procedure in an operative setting<br>☐ Planned interventional radiology procedure<br>☐ Central line placement<br>☐ Concern resulting in ICU admission	☐ Urgent or emergent surgical procedure	☐ Single organ failure secondary to endocrine dysfunction or sequelae	☐ Multi organ failure secondary to endocrine dysfunction or sequelae	☐ Death attributed to endocrine dysfunction or sequelae	☐ Temporary (< 12 months)<br>☐ Permanent (> 12 months)<br>☐ Unknown
Flap Complication<br><br>☐ Local<br>☐ Regional<br>☐ Free flap<br>-------------------<br>☐ Wound dehiscence<br>☐ Venous congestion<br>☐ Arterial compromise<br>-------------------<br>☐ Partial flap loss – any loss of part of the flap with continued perfusion of the flap<br>☐ Complete flap loss – discontinued perfusion of the flap	☐ Oral medications<br>☐ Topical medications<br>☐ Leech therapy<br>☐ Concern resulting in Emergency room evaluation	☐ Wound exploration ± debridement ± packing in non-operative setting<br>☐ Wound vacuum placement<br>☐ IV medications<br>☐ Hyperbaric oxygen therapy<br>☐ Concern resulting in hospital admission	☐ Planned surgical procedure in an operative setting<br>☐ Planned interventional radiology procedure<br>☐ Concern resulting in ICU admission	☐ Urgent or emergent surgical procedure	☐ Single organ failure secondary to flap complication or sequelae<br>☐ Complete flap loss	☐ Multi organ failure secondary to flap complication or sequelae	☐ Death attributed to flap complication or sequelae	☐ Temporary (< 12 months)<br>☐ Permanent (> 12 months)<br>☐ Unknown

© 2025 Hidalgo CM et al. *JAMA Network Open*.

-------------------<br>☐ Microvascular anastomosis revision<br>☐ Salvage with local flap<br>☐ Salvage with regional flap<br>☐ Salvage with free flap								
Hemorrhage	☐ Topical medications<br>☐ Conservative cares (e.g., pressure, ice water gargles)<br>☐ Concern resulting in Emergency room evaluation	☐ Wound exploration ± packing in non-operative setting<br>☐ Wound cauterization in non-operative setting<br>☐ IV medications<br>☐ Blood transfusion/factor replacement<br>☐ Concern resulting in hospital admission	☐ Planned surgical procedure in an operative setting<br>☐ Planned interventional radiology procedure<br>☐ Concern resulting in ICU admission	☐ Urgent or emergent surgical procedure<br>☐ Urgent or emergent interventional radiology procedure	☐ Single organ failure secondary to hemorrhage or sequelae	☐ Multi organ failure secondary to hemorrhage or sequelae	☐ Death attributed to hemorrhage or sequelae	☐ Temporary (< 12 months)<br>☐ Permanent (> 12 months)<br>☐ Unknown
Post-operative fluid or air collection excluding chyle, infection/purulence, or cerebrospinal fluid (CSF):<br>☐ Hematoma<br>☐ Seroma<br>☐ Saliva<br>☐ Fistula<br>☐ Air trapping	☐ Conservative management (i.e., outpatient observation, pressure dressing)<br>☐ High concentration 02 delivery	☐ Wound exploration ± packing in non-operative setting<br>☐ Nasogastric tube placement<br>☐ IV medications<br>☐ Concern resulting in	☐ Planned surgical procedure in an operative setting<br>☐ Planned interventional radiology procedure<br>☐ PEG, G, or J tube placement	☐ Urgent or emergent surgical procedure	☐ Single organ failure secondary to post-op fluid collection or sequelae	☐ Multi-organ failure secondary to post-op fluid collection or sequelae	☐ Death attributed to post-op fluid collection or sequelae	☐ Temporary (< 12 months)<br>☐ Permanent (> 12 months)<br>☐ Unknown

© 2025 Hidalgo CM et al. *JAMA Network Open*.

	☐ Oral medications<br>☐ Topical medications<br>☐ Modification of diet<br>☐ Needle aspiration<br>☐ Concern resulting in Emergency room evaluation	hospital admission	☐ Total parenteral nutrition (TPN)<br>☐ Concern resulting in ICU admission					
Motor nerve injury<br>☐ CN II<br>☐ CN III<br>☐ CN IV<br>☐ CN V (V3)<br>☐ CN VI<br>☐ CN VII (HB > II/VI)<br>☐ CN X<br>☐ CN XI<br>☐ CN XII<br>☐ Brachial plexus<br>☐ Other motor n.	☐ Conservative management (i.e., observation, eye bubble, artificial tears)<br>☐ Oral medications<br>☐ Modification of diet<br>☐ Physical/occupational/speech therapy<br>☐ Concern resulting in Emergency room evaluation	☐ IV medications<br>☐ Nasogastric tube placement<br>☐ Concern resulting in hospital admission	☐ Planned surgical procedure in an operative setting<br>☐ Planned interventional radiology procedure<br>☐ Concern resulting in ICU admission	☐ Urgent or emergent surgical procedure	☐ Single organ failure secondary to motor nerve injury	☐ Multi-organ failure secondary to motor nerve injury	☐ Death attributed to motor nerve injury	☐ Temporary (< 12 months)<br>☐ Permanent (> 12 months)<br>☐ Unknown
Sensory or autonomic nerve injury<br>☐ CN I<br>☐ CN V<br>☐ CN VIII<br>☐ CN IX<br>☐ CN X<br>☐ Other sensory nerve, please specify:	☐ Conservative management<br>☐ Oral medications<br>☐ Local injection<br>☐ Concern resulting in Emergency	☐ IV medications<br>☐ Nerve block<br>☐ Concern resulting in hospital admission	☐ Planned surgical procedure in an operative setting<br>☐ Planned interventional radiology procedure	☐ Urgent or emergent surgical procedure	☐ Single organ failure secondary to sensory nerve injury	☐ Multi-organ failure secondary to sensory nerve injury	☐ Death attributed to sensory nerve injury	☐ Temporary (< 12 months)<br>☐ Permanent (> 12 months)<br>☐ Unknown

© 2025 Hidalgo CM et al. *JAMA Network Open*.

☐ Sympathetic chain	room evaluation		☐ Nerve block or pump requiring sedation<br>☐ Concern resulting in ICU admission					
Surgical site infection (SSI)	☐ Oral medications<br>☐ Topical medications<br>☐ Superficial wound dressings<br>☐ Needle aspiration<br>☐ Concern resulting in Emergency room evaluation	☐ IV antibiotics<br>☐ Wound exploration ± packing in non-operative setting<br>☐ Concern resulting in hospital admission	☐ Planned surgical procedure in an operative setting<br>☐ Planned interventional radiology procedure<br>☐ Concern resulting in ICU admission	☐ Urgent or emergent surgical procedure	☐ Single organ failure secondary to SSI or sequelae	☐ Multi-organ failure secondary to SSI or sequelae	☐ Death attributed to SSI or sequelae	☐ Temporary (< 12 months)<br>☐ Permanent (> 12 months)<br>☐ Unknown
Vascular injury	☐ Conservative management<br>☐ Oral medications<br>☐ Topical medications<br>☐ Concern resulting in Emergency room evaluation	☐ IV medications<br>☐ Wound exploration and cauterization and/or packing in a non-operative setting<br>☐ Concern resulting in hospital admission	☐ Planned surgical procedure in an operative setting<br>☐ Planned interventional radiology procedure<br>☐ Concern resulting in ICU admission	☐ Urgent or emergent surgical procedure<br>☐ Urgent or emergent interventional radiology procedure	☐ Single organ failure secondary to vascular injury or sequelae	☐ Multi-organ failure secondary to vascular injury or sequelae<br>☐ Life-threatening vascular injury	☐ Death attributed to vascular injury or sequelae	☐ Temporary (< 12 months)<br>☐ Permanent (> 12 months)<br>☐ Unknown
Device or Implant Concern	☐ Concern resulting in Emergency room evaluation	☐ Planned procedure performed in non-operative setting	☐ Planned surgical procedure in an operative setting	☐ Urgent or emergent surgical procedure	☐ Single organ failure due to device or implant	☐ Multi-organ failure secondary to device or implant	☐ Death attributed to device or implant	☐ Temporary (< 12 months)<br>☐ Permanent (> 12 months)<br>☐ Unknown

© 2025 Hidalgo CM et al. *JAMA Network Open*.

		☐ Concern resulting in hospital admission	☐ Planned interventional radiology procedure<br>☐ Concern resulting in ICU admission					
**Medical**								
Cardiovascular<br>☐ Hypertension<br>☐ Hypotension<br>☐ Myocardial infarction<br>☐ Angina<br>☐ Atrial fibrillation<br>☐ Arrythmia<br>☐ Heart failure<br>☐ Other, please specify:	☐ Observation<br>☐ Oral medications<br>☐ Topical medications<br>☐ Concern resulting in Emergency room evaluation	☐ IV medications<br>☐ IV resuscitation<br>☐ Concern resulting in hospital admission	☐ Planned surgical procedure in an operative setting<br>☐ Planned interventional radiology procedure<br>☐ Concern resulting in ICU admission	☐ Urgent or emergent surgical procedure	☐ Single organ failure secondary to cardiovascular toxicity or sequelae	☐ Multi-organ failure secondary to cardiovascular toxicity or sequelae	☐ Death secondary to cardiovascular toxicity or sequelae	☐ Temporary (< 12 months)<br>☐ Permanent (> 12 months)<br>☐ Unknown
Respiratory<br>☐ Atelectasis<br>☐ Bronchitis/Pneumonitis<br>☐ Pneumonia<br>☐ Pulmonary embolus<br>☐ Reactive airway/COPD exacerbation<br>☐ Other, please specify:	☐ Supplemental oxygen<br>☐ Oral medications<br>☐ Inhaled medications<br>☐ Nebulized medications<br>☐ Unplanned endoscopic exam<br>☐ Concern resulting in Emergency room evaluation	☐ IV medications<br>☐ Non-invasive positive pressure<br>☐ Nasogastric tube placement<br>☐ Concern resulting in hospital admission	☐ Planned surgical procedure in an operative setting<br>☐ Planned interventional radiology procedure<br>☐ Unexpected intubation<br>☐ Unexpected mechanical ventilation < 24 hours<br>☐ Concern resulting in ICU admission	☐ Mechanical ventilation > 24 hours<br>☐ Urgent or emergent surgical procedure	☐ Single organ failure secondary to respiratory toxicity or sequelae	☐ Multi-organ failure secondary to respiratory toxicity or sequelae	☐ Death secondary to respiratory toxicity or sequelae	☐ Temporary (< 12 months)<br>☐ Permanent (> 12 months)<br>☐ Unknown

© 2025 Hidalgo CM et al. *JAMA Network Open*.

Neurologic<br>☐ Alcohol withdrawal<br>☐ Altered mental status (delirium) or worsening of dementia<br>☐ Cerebrovascular accident or stroke<br>☐ Substance withdrawal<br>☐ Seizure<br>☐ Transient ischemic attack<br>☐ Other, please specify:	☐ Supplemental oxygen<br>☐ Oral medications<br>☐ Physical/occupational/speech/neuro rehabilitation<br>☐ Concern resulting in Emergency room evaluation	☐ IV medications<br>☐ Nasogastric tube placement, NPO, enteral nutrition only<br>☐ Concern resulting in hospital admission	☐ Planned surgical procedure in an operative setting<br>☐ Planned interventional radiology procedure<br>☐ PEG, G, or J tube placement<br>☐ Total parenteral nutrition (TPN)<br>☐ Unexpected intubation<br>☐ Unplanned mechanical ventilation < 24 hours<br>☐ Concern resulting in ICU admission	☐ Unplanned mechanical ventilation > 24 hours<br>☐ Urgent or emergent surgical procedure	☐ Single organ failure secondary to neurologic toxicity or sequelae	☐ Multi-organ failure secondary to neurologic toxicity or sequelae	☐ Death secondary to neurologic toxicity or sequelae	☐ Temporary (< 12 months)<br>☐ Permanent (> 12 months)<br>☐ Unknown
Hematologic/Vascular<br>☐ Deep venous thrombosis<br>☐ Anemia<br>☐ Other, please specify:	☐ Supplemental oxygen<br>☐ Oral medications<br>☐ Concern resulting in Emergency room evaluation	☐ IV medications<br>☐ Blood transfusion<br>☐ Concern resulting in hospital admission	☐ Planned surgical procedure in an operative setting<br>☐ Planned interventional radiology procedure<br>☐ Concern resulting in ICU admission	☐ Urgent or emergent surgical procedure	☐ Single organ failure secondary to hematologic/vascular toxicity or sequelae	☐ Multi-organ failure secondary to hematologic/vascular toxicity or sequelae	☐ Death secondary to hematologic/vascular toxicity or sequelae	☐ Temporary (< 12 months)<br>☐ Permanent (> 12 months)<br>☐ Unknown
Genitourinary (GU)/Renal	☐ Oral medications<br>☐ Concern resulting in	☐ IV medications	☐ Planned surgical procedure in an	☐ Urgent or emergent surgical procedure	☐ Single organ failure secondary	☐ Multi-organ failure secondary to	☐ Death secondary to GU/renal	☐ Temporary (< 12 months)

© 2025 Hidalgo CM et al. *JAMA Network Open*.

☐ Urinary tract infection<br>☐ Acute kidney injury<br>☐ Urinary retention<br>☐ Traumatic foley insertion necessitating prolonged catheter<br>☐ Other, please specify:	Emergency room evaluation	☐ Foley catheter placement<br>☐ Bladder irrigation<br>☐ Electrolyte replacement<br>☐ Concern resulting in hospital admission	operative setting<br>☐ Planned interventional radiology procedure<br>☐ Concern resulting in ICU admission		to GU/renal toxicity or sequelae<br>☐ Dialysis	genitourinary/renal toxicity or sequelae	toxicity or sequelae	☐ Permanent (> 12 months)<br>☐ Unknown
Infectious	☐ Oral medications<br>☐ Concern resulting in Emergency room evaluation	☐ IV medications<br>☐ Blood cultures<br>☐ Concern resulting in hospital admission	☐ Planned surgical procedure in an operative setting<br>☐ Planned interventional radiology procedure<br>☐ Concern resulting in ICU admission	☐ Urgent or emergent surgical procedure	☐ Single organ failure secondary to infectious toxicity or sequelae	☐ Multi-organ failure secondary to infectious toxicity or sequelae	☐ Death secondary to infectious toxicity or sequelae	☐ Temporary (< 12 months)<br>☐ Permanent (> 12 months)<br>☐ Unknown
Gastrointestinal (GI)<br><br>☐ Nausea<br>☐ Ileus<br>☐ Intestinal obstruction<br>☐ Intestinal ischemia<br>☐ Constipation<br>☐ Other, please specify:	☐ Oral medications<br>☐ Suppository medications<br>☐ Modification of diet or fluid intake<br>☐ Enema<br>☐ Concern resulting in Emergency room evaluation	☐ IV medications<br>☐ Nasogastric feeding tube placement<br>☐ Gastrointestinal decompression via gastric or rectal tube<br>☐ Concern resulting in hospital admission	☐ Planned surgical procedure in an operative setting<br>☐ Planned interventional radiology procedure<br>☐ PEG, G, or J tube placement<br>☐ Total parenteral nutrition (TPN)	☐ Urgent or emergent surgical procedure<br>☐ Creation of a temporary ostomy	☐ Single organ failure secondary to GI toxicity or sequelae<br>☐ Creation of a permanent ostomy	☐ Multi-organ failure secondary to GI toxicity or sequelae	☐ Death secondary to GI toxicity or sequelae	☐ Temporary (< 12 months)<br>☐ Permanent (> 12 months)<br>☐ Unknown

© 2025 Hidalgo CM et al. *JAMA Network Open*.

			☐ Concern resulting in ICU admission					
**Comprehensive Complication Index (CCI)**								
Overall score*								

Oto-HNS, Otolaryngology-Head and Neck Surgery; CDC, Clavien-Dindo Classification
[a] *Intervention for toxicity may occur > 30 days following diagnosis. E.g., Gracilis free flap for facial reanimation may be done 6 months post operatively, but the CN VII injury was diagnosed < 30 days post-operatively.*
**Calculation: CCI® Calculator*

© 2025 Hidalgo CM et al. *JAMA Network Open*.

eAppendix. Supplemental Clinical Vignettes

Vignette 1 Scenario
A 13-year-old, otherwise healthy male undergoes routine tonsillectomy for recurrent tonsillitis. In the postoperative care unit, he develops stridor but no oxygen desaturation. On exam, the surgeon notes mild edema of the cords. His exam and vitals are otherwise within normal limits. The surgeon recommends a dose of oral steroids, and his symptoms resolve. He is monitored closely and then discharged to home on a 3-day steroid taper. The remainder of the patient's course is within normal limits.

Vignette 2 Scenario
A 65-year-old female undergoes bilateral functional endoscopic sinus surgery for chronic rhinosinusitis with nasal polyposis. Surgery is successful and she is sent home on the surgeon's usual post-operative analgesic regimen of acetaminophen and ibuprofen. On postoperative day 3, the patient presents to the local emergency department with severe pain. The patient is afebrile, there is no purulence on exam, and her computed tomography (CT) scan in the emergency department does not reveal any concerning pathology. The patient is given an IV dose of hydromorphone and sent home with a 3-day prescription of oral narcotics. The patient reports no longer needing any analgesic medications at her two-week follow-up visit.

Vignette 3 Scenario
A 78-year-old gentleman with a 50-pack year smoking history presents with an advanced laryngeal cancer and cervical lymphadenopathy. The tumor board recommendation after completion of his work up is for total laryngectomy and bilateral neck dissection. The procedure occurs without complication. On post-operative day (POD) 1, enteral feeds are started and by the afternoon, 600 cc of milky fluid is charted from the patient's left neck drain. The patient is made immediately NPO and started on subcutaneous octreotide. The drain is placed on bulb suction, pressure dressings are applied, the patient is placed on a bowel regimen, and activity is limited. The drain output continues to remain > 500 cc/24 hr. A thoracic duct ligation is performed by thoracic surgery. The patient is started on a medium chain fatty acid diet. Drain output drops significantly, and the patient is discharged on POD 8 with a drain. The drain was pulled in the outpatient clinic on POD 10. The patient goes on to complete adjuvant chemoradiation therapy without delay.

Vignette 4 Scenario
A 41-year-old female presents with a 3 cm right-sided vestibular schwannoma. This has resulted in progressive neurologic sequelae and retrosigmoid surgical approach for attempt at gross total resection is recommended. There are no concerns intraoperatively. On postoperative day (POD) 2 a fluid accumulation is noted under the wound and on POD 3, clear fluid is noted to be leaking from the incision. This is positive for beta-2 transferrin. The patient is otherwise neurologically intact, and vitals are stable. The patient is placed on bed rest, made NPO, and scheduled for surgical intervention the next day. The re-exploration results in resolution of the cerebrospinal fluid (CSF) leak, and the patient is discharged home on POD 5.

Vignette 5 Scenario
A 41-year-old woman undergoes a functional septorhinoplasty for nasal obstruction. She is otherwise healthy and tolerates surgery without issue. She is discharged home in stable condition on postoperative day (POD) 0. One-week and two-week follow-up visits demonstrate excellent healing and patent nasal passages. The patient reports she is no longer needing analgesics at her two-week post-op visit. You receive a notice from family that on POD 20, the patient was found dead in her home. There was no obvious cause of death based on the emergency medicine notes and her toxicology studies were unrevealing. The family declines an autopsy.

Vignette 6 Scenario
A 74-year-old male presents with a large Zenker's diverticulum. A transoral approach is unsuccessful and therefore the surgeon converts to an open approach. This results in complete cricopharyngeal myotomy and the redundant diverticulum tissue is surgically removed. The patient is discharged home and instructed to advance their diet from non-particulate clears for 2 days, to particulate clears for 2 days, to mechanical soft for 1 week, to a normal diet per routine. The patient does well and returns one month later with a much-improved swallow study and improved quality of life.

© 2025 Hidalgo CM et al. *JAMA Network Open*.

Vignette 7 Scenario
A 31-year-old female presents with a bulky thyroid nodule and highly suspicious central neck lymphadenopathy. Biopsy is consistent with papillary thyroid carcinoma. She undergoes a total thyroidectomy and central neck dissection. She is admitted to the hospital for routine calcium monitoring and drain management. Her 6-hour post-op parathyroid hormone (PTH) is undetectable, and her calcium is just below the normal range. Oral supplementation is started the evening of surgery. On post-operative day (POD) 1, she remains hypocalcemic and becomes symptomatic. She is admitted to the intensive care unit (ICU) for IV calcium supplementation and cardiac monitoring. After 24 hours, her calcium stabilizes, and she is moved back to the general floor and then discharged to home on POD 3. She is maintained on oral calcium and vitamin D supplementation in the outpatient setting. At her last follow-up 2 years post-op, she still requires oral calcium and vitamin D supplementation for undetectable PTH. She has no sign of recurrent disease.

Vignette 8 Scenario
A 61-year-old female presents with a long-standing basal cell carcinoma that has invaded her orbit, maxilla, and intranasal cavity. She undergoes a large operation requiring orbital exenteration, craniotomy to clear dural margins, near total rhinectomy, and maxillectomy. She is reconstructed with an anterolateral thigh flap. On postoperative day (POD) 2, the intraoral portion of the flap becomes congested, and fullness is noted in the patient's neck consistent with a hematoma. Intraoral flap prick reveals sluggish dark blood. The patient is rushed to the operating room for re-exploration. The hematoma is evacuated and the flap pedicle is investigated. There is a strong doppler signal from both the artery and the vein. The flap is pricked and shows dark blood, but it is brisker, and the flap becomes less congested. The wound is closed, and the patient is brought back to the general floor. The remainder of her hospital stay is uneventful.

Vignette 9 Scenario
A 68-year-old male with essential tremor presents with a fungus ball located in the left maxillary sinus. He successfully undergoes left maxillary antrostomy for removal and treatment. He does well and is discharged home the same day on oral antibiotics, acetaminophen, and ibuprofen. He presents to an emergency department later that evening with profuse left-sided epistaxis. He is unable to control his airway and is intubated in the emergency department and taken urgently to the operating room for endoscopic investigation. His sphenopalatine artery is ligated and the bleeding resolves. He has some packing placed and is observed overnight in the hospital. He is discharged the next day. The remainder of his postoperative course is uneventful.

Vignette 10 Scenario
A 44-year-old male undergoes superficial parotidectomy via an extracapsular dissection for a benign pleomorphic adenoma. He is discharged the same day without a drain. He does well postoperatively but calls in 7 days later with concerns that every time he eats, he gets clear drainage from his incision. His exam is consistent with a sialocele. He is prescribed scopolamine patches and conservative care. The sialocele resolves within 2 weeks. The remainder of his postoperative care is uneventful.

Vignette 11 Scenario
A 53-year-old female undergoes cochlear implantation on the left side. The surgery is challenging secondary to a sclerotic mastoid and evidence of prior chronic ear disease. The implant appears in good position and excellent feedback is noted. In the recovery unit, the patient is noted to have House Brackmann (HB) grade III/VI facial nerve function on the left. The surgeon feels confident that the facial nerve was intact at the end of the operation and a steroid burst and taper is prescribed. On postoperative day (POD) 6, the patient notes improved facial nerve function and by POD 14 she is noted to have HB I/VI. She has excellent function of her cochlear implant.

Vignette 12 Scenario
An 81-year-old female presented with progressive vision changes and is diagnosed with a retro-orbital hemangioma. An endoscopic resection is performed with a joint neurosurgical and otolaryngology team. The surgical team performs a subtotal resection in order to protect the patient's orbital nerve. The case is completed without incident. In the post-anesthesia care unit, the patient complains of decreased vision in the affected eye. This progressively worsens over the subsequent 24 hours. CT scan is unrevealing. Ophthalmology consult reveals vision to light only and no reversable pathology on exam. The patient is taken back for an urgent surgical re-exploration. The orbital nerve is felt to be intact, and no other correctable pathology is identified. She is placed on a steroid burst and taper. At her 3-month postoperative visit, she has no vision in the left eye.

© 2025 Hidalgo CM et al. *JAMA Network Open*.

Vignette 13 Scenario
A 56-year-old type I diabetic undergoes submandibular gland excision for recurrent sialoadenitis. The patient does well postoperatively and is discharged to home. On postoperative day (POD) 7, he calls in concerned about peri-incisional erythema and systemic body aches. He is evaluated in clinic and found to have an abscess. This is opened in clinic revealing 10 cc of purulent material. The wound is copiously irrigated and packed with antibiotic gauze. He is placed on oral antibiotics, recommended tight glycemic control, and is instructed to pack his wound twice daily. By two weeks, he is no longer able to pack the wound as it has healed quite well. He completes his antibiotics and has no further sequelae.

Vignette 14 Scenario
A 36-year-old male presents for evaluation of a displaced septal button. The button was inserted 6 days earlier in the operating room for a 1 cm nasal septal perforation. The patient is prescribed an oral sedative and tolerates replacement of his septal button in-clinic with local anesthesia. He is discharged with no change to his postoperative recommendations.

Vignette 15 Scenario
A 43-year-old female presents for an enlarging carotid body paraganglioma. She undergoes work up and it is found to be a Shamblin type I (not encasing any component of the carotid system) and is nonsecretory. She is taken to the operating room and undergoes resection. There are no intraoperative or postoperative complications. She undergoes routine postoperative imaging 3 weeks later and is noted to have a large pseudoaneurysm along the external carotid artery within the operative bed. She is scheduled for a stent placement across the aneurysm by interventional radiology. She tolerates this procedure well and has no long-term sequelae.

Vignette 16 Scenario
A 68-year-old woman undergoes neck exploration and resection of a large benign lipoma. She tolerates this procedure well. A drain is placed, and she is brought to the floor for observation following surgery. Nursing notes tachycardia and an electrocardiogram (EKG) reveals atrial fibrillation (AFib) with rapid ventricular rate. She has no history of AFib. Cardiology recommends cardioversion within 24 hours and rate control with a beta-blocker. She successfully undergoes cardioversion and is stable in the hospital for 24 hours. She is discharged home with close cardiology follow-up.

Vignette 17 Scenario
A 52-year-old male presents with a cT4 oral cavity cancer. He undergoes partial glossectomy, fibular free flap reconstruction and bilateral neck dissection. He has a tracheostomy and nasogastric tube placed. On postoperative day 3 he is noted to have a creatinine of 3.7 and becomes symptomatic. Urgent urology workup reveals a critical renal artery stenosis and acute kidney injury stage 4. He undergoes stent placement with interventional radiology and requires dialysis. He is discharged home on dialysis with close urology follow-up. At 6 months post-op, he has completed radiation alone and his renal function has improved. He no longer needs dialysis, and his creatinine has normalized.

Vignette 18 Scenario
A 74-year-old male undergoes right total parotidectomy, lateral temporal bone resection, neck dissection, and right anterolateral thigh free flap reconstruction for a locally advanced squamous cell carcinoma. In the postoperative care unit, it is noted that he has no motor function in his left arm or leg. An urgent CT scan reveals a massive left-sided hemorrhagic stroke. The patient is monitored in the ICU and transferred to a neurologic rehabilitation unit for recovery. He is unable to undergo adjuvant therapy and ultimately is diagnosed with metastatic disease 6 months later. He is transitioned to palliative care and dies 9 months after his initial operation.

Vignette 19 Scenario
A 48-year-old obese female undergoes hypoglossal nerve stimulator placement for moderate obstructive sleep apnea. Her surgery was uneventful, and she was dismissed on postoperative day (POD) 1. She presents to the emergency room on POD 4 complaining of pain, tenderness, and warmth of her right calf. Physical examination and duplex ultrasonography reveal a deep venous thrombosis (DVT). She is admitted to the hospital and treated with low molecular weight heparin. She is dismissed after 24 hours and ultimately is transitioned to oral warfarin therapy and compression stockings. After 6 months repeat imaging reveals resolution of her DVT and her therapy is discontinued.

© 2025 Hidalgo CM et al. *JAMA Network Open*.

Vignette 20 Scenario
A 26-year-old healthy female undergoes a routine otoplasty for auricularis prominauris. Postoperatively she has intractable nausea and vomiting. She remains in the hospital for overnight observation requiring IV Promethazine, IV Ondansetron and IV Dexamethasone in an effort to combat her symptoms. The following day, her nausea and vomiting resolve and she is dismissed from the hospital. At her 2-week follow-up she has no further clinical concerns and the remainder of her postoperative course is unremarkable.

Vignette 21 Scenario
A 56-year-old alcoholic male undergoes total laryngectomy, bilateral neck dissections, and pectoralis major regional flap reconstruction for advanced laryngeal carcinoma. On postoperative day (POD) 1, he begins experiencing tachycardia, hypertension, and disorientation. He is treated with IV benzodiazepines with minimal benefit. The patient becomes tremulous, further disoriented, and ultimately suffers a grand mal seizure. He is urgently intubated and treated with multi-agent therapy for alcohol withdrawal syndrome. After 3 days of IV therapy, mechanical ventilation, and supportive cares, he is weaned from the ventilator, extubated, and managed further with enteral and IV support. The remainder of his hospitalization goes well, and he is ultimately discharged on POD 11.

Vignette 22 Scenario
A 61-year-old female with a history of long-standing idiopathic right vocal cord paralysis undergoes a thyroplasty for phonation improvement. Her operation takes approximately 2.5 hours to perform, and she is given approximately 3 liters of fluid intraoperatively. While in the post-operative anesthesia care unit the patient becomes stridorous and complains about rapidly progressing dyspnea. She is given IV dexamethasone, yet her saturations begin to drop. The patient quickly becomes unresponsive and attempts at intubation are unsuccessful due to extensive glottic edema. The surgeon performs a bedside surgical tracheostomy and quickly secures the airway. The patient is placed on a ventilator for 12 hours with IV dexamethasone. She is ultimately extubated and suffers no neurologic sequelae. She is dismissed with her tracheostomy tube on postoperative day 3. One month later endoscopic evaluation of her larynx reveals an adequate airway, and she is decannulated.

Vignette 23 Scenario
A 39-year-old healthy female undergoes hemithyroidectomy for a follicular adenoma. She is dismissed home on postoperative day (POD) 0 without a drain and with the recommendation to take over-the-counter medications for pain. On POD 2 she presents to the emergency room with increasing anterior neck pain. Physical examination shows a flat, non-erythematous, nonfluctuant, and non ecchymotic anterior neck incision. Ultrasound reveals postoperative changes without evidence of a fluid collection. She is given a prescription for oral tramadol to be taken every 6 hours as needed for pain. A follow-up phone call 24 hours later reveals that the patient's pain is now controlled.

Vignette 24 Scenario
A 17-year-old overweight male presents to the Otolaryngology clinic with a fluctuant left neck mass. He had similar cervical findings at age 5 and age 12 for which he underwent operative neck explorations at an outside institution. Revision left neck surgery is performed in attempt to remove a formidable cervical lymphovenous malformation. The procedure involved removal of levels 3 and 4 of the left neck as well as the left hemi thyroid and a cuff of the sternocleidomastoid muscle. At the end of the case, hemostasis was obtained and a Valsalva maneuver revealed no evidence of left neck fluid. A suction drain was placed, and the patient was admitted to the hospital for observation overnight. After eating a cheeseburger on postoperative day (POD) 1, he was noted to have a subtle amount of milky fluid in his drain. The fluid is sent for analysis and found to be positive for chylomicrons. His drain is placed on bulb suction, he is prescribed a medium chain triglyceride diet, and his activity is limited. On POD 2 he is dismissed from the hospital with his drain in place. The patient's mother calls on POD 5 stating that the drain output has completely diminished, and that there is no longer a milky tinge to the fluid in the drain. The patient is seen that afternoon and his drain is pulled. The remainder of his postoperative course is unremarkable.

Vignette 25 Scenario
A 56-year-old male with a history of coronary artery disease undergoes total parotidectomy for low-grade acinic cell carcinoma. On postoperative day (POD) 1, he is found to have rapid evolution of a hematoma. He undergoes uneventful operative exploration and hematoma evacuation. In the postanesthesia care unit, he becomes tachycardic, diaphoretic, and complains of crushing substernal chest pain. A cardiac workup demonstrates substantial myocardial ischemia consistent with a STEMI. He undergoes emergent percutaneous coronary intervention with balloon

© 2025 Hidalgo CM et al. *JAMA Network Open*.

angioplasty. He recovers uneventfully from this procedure. The remainder of his postoperative course is uneventful, and he is dismissed from the hospital on POD 6 with appropriate oncologic and cardiac follow-up.

Vignette 26 Scenario

A 51-year-old female undergoes elective rhytidectomy for aging face. The procedure is uneventful and without identified complication. The patient, however, is noted to have complete ipsilateral brow paralysis on postoperative day (POD) 1. Reassurance is provided and plans are made for follow-up. The patient presents to the emergency room on POD 5 with extensive peri-incisional erythema, warmth, and tenderness. She is diagnosed with a surgical site infection and admitted for IV antibiotic therapy. She is subsequently transitioned to oral antibiotics and dismissed from the hospital 2 days later after symptomatic and physical exam improvement. Clinical follow-up 2 weeks later reveals resolution of her infection but persistent complete brow paralysis. She is treated with contralateral forehead Botox injection. Unfortunately, she has persistent brow paralysis at 1 year and plans are made for a brow lift.

Vignette 27 Scenario

A 49-year-old female was diagnosed with bilateral otosclerosis. She underwent an uneventful stapedotomy on the right side. Postoperatively, she complained of a sensation of significant dizziness/unsteadiness and was noted to have nystagmus beating to the left. 36 hours after dismissal she presented to the emergency room with increasing pain and subjective decline in her hearing. Exam at that time showed canal packing in place, persistent nystagmus, but no signs of infection. She was treated with intramuscular tramadol and given a small supply of oxycodone for pain management. Over the next 5 weeks, the patient developed progressive hearing loss on the right side accompanied with dizziness. She was treated with antibiotics and steroids, yet her hearing loss progressed to a profound loss (dead ear). She underwent revision stapedotomy, during which abundant granulation tissue was found to be filling the entire oval window. Biopsy was compatible with reparative granuloma. Eight weeks later, the patient was free from dizziness but her hearing did not recover.

Vignette 28 Scenario

A 68-year-old healthy male undergoes revision endoscopic sinus surgery for extensive nasal polyposis. On postoperative day (POD) 0, he is unable to void and undergoes in and out catheterization on two occasions and is started on PO terazosin. He is admitted overnight for observation. The following morning a foley catheter is placed due to persistent urinary retention. As the patient is preparing for dismissal, he develops significant bilateral epistaxis. Bedside measures fail to control the bleeding, and the patient is swiftly taken to the operating room where he undergoes endonasal cauterization and intranasal packing. He recovers uneventfully and is dismissed on POD 3 with a foley catheter in place. He follows up with the urology team for catheter removal 1 week later. The remainder of his postoperative course is uneventful.

Vignette 29 Scenario

A 68-year-old female with a history of post-polio syndrome, dyslipidemia, and coronary artery disease presents with a rapidly enlarging anterior neck mass. Fine needle aspiration biopsy reveals dedifferentiated papillary thyroid carcinoma. A total thyroidectomy with synchronous bilateral central compartment neck dissection is performed. Her left recurrent nerve is inadvertently sacrificed. Postoperatively, the patient struggles with secretion management and aspiration. A nasogastric feeding tube is placed for nutritional support. By postoperative day 3, her work of breathing increases and she is unable to maintain her saturations. A chest x-ray shows bilateral aspiration pneumonia. IV antibiotics are initiated, but her condition rapidly deteriorates requiring urgent intubation and transfer to the intensive care unit. She medically declines over the next several days, becoming septic, developing acute renal failure, and suffering a mild myocardial infarction. After a tumultuous 26 days in the hospital during which time she undergoes a tracheostomy and PEG tube placement, she is discharged to a high acuity skilled nursing facility.

Vignette 30 Scenario

A 46-year-old male undergoes transoral robotic surgery and neck dissection for a pT2N1M0 human papillomavirus (HPV) positive squamous cell carcinoma of the right tonsil. While in the hospital, he struggles with swallowing but repeatedly declines a feeding tube. At his 2-week postoperative visit he continues to complain of moderate pain and persistent, yet improving, dysphagia and odynophagia. He has lost 30 pounds, going from 210 pounds preoperatively to 180 pounds. The patient refuses nutritional counseling and defers adjuvant therapy recommendations. At his 1-year follow-up he weighs 182 pounds and is tumor free on clinical and radiographic imaging. He notes that his swallowing has returned to baseline.

© 2025 Hidalgo CM et al. *JAMA Network Open*.

Vignette 31 Scenario
A 15-year-old female has a Sistrunk procedure performed for an infected thyroglossal duct cyst. Her father contacts the surgical team shortly after hospital dismissal, noting that his daughter has intractable nausea and vomiting. Oral antiemetic therapy is recommended but provides little benefit. The patient presents to the emergency room where her physical examination reveals an intact anterior neck incision, soft neck with no evidence of fluid collection, but tongue deviation to the left. She is treated with multi-agent IV antiemetic therapy with good benefit. At her 6-week follow-up she continues to note leftward tongue deviation and mild dysarthria. It is recommended that this finding be observed and that she sees speech therapy. When evaluated at follow-up 1 year later she is found to have left hemitongue atrophy, left tongue fasciculations, and persistent subjective dysarthria.

Vignette 32 Scenario
A 38-year-old male with a respiratory disturbance index (RDI) of 96 is found to have severe lingual tonsil hypertrophy. He undergoes transoral robotic lingual tonsillectomy and is dismissed on postoperative day (POD) 1. He presents to the emergency department on POD 3 complaining of shortness of breath, severe dysphagia, nausea, and 9/10 pain despite triple agent PO analgesia. He is found to have oxygen saturations ranging from 70-80% and significant supraglottic edema on evaluation with flexible bedside nasopharyngolaryngoscopy. He is placed on 2 liters of oxygen, and given IV morphine, IV dexamethasone, and IV promethazine with mild symptom improvement. He is admitted for overnight observation. The following day, he is found unresponsive in his hospital bed. All attempts at resuscitation fail and he is pronounced dead.

Vignette 33 Scenario
A 23-year-old female with a nasal deformity and airway obstruction undergoes a septorhinoplasty. She presents to the clinic on postoperative day 4 complaining of increasing nasal pain and a low-grade fever. Her intranasal splints are removed, and she is found to have a septal abscess. Pain precludes in office management, and she is taken to the operating room for incision and drainage of the abscess. She is dismissed on a 10-day course of 875 mg twice daily amoxicillin/clavulanate potassium, intranasal mupirocin, and saline irrigation. Repeat evaluation in 1 week reveals abscess resolution but a 5 mm anterior nasal septal perforation, which the patient chooses to observe. Evaluation 6 months postoperatively reveals enlargement of the perforation and the patient complains of intractable nasal crusting. She elects to undergo septal perforation repair the following week which occurs uneventfully.

Vignette 34 Scenario
A 78-year-old male smoker with a history of peripheral vascular disease and hypertension is diagnosed with a cT4 oral cavity squamous cell carcinoma invading his mandible. Lower extremity imaging reveals narrow caliber 3 vessel runoff bilaterally. The patient undergoes surgical therapy for his malignancy which includes fibular free flap reconstruction. On postoperative day (POD) 2 the patient complains of increasing donor site leg pain. On exam, his foot is cold to palpation with absent anterior and posterior pulses. His leg wound is urgently opened at bedside without clinical improvement. A computed tomography angiography (CTA) reveals complete lower extremity arterial occlusion. Vascular surgery is emergently consulted, and the patient undergoes several operations over the next 5 days in an attempt to re-establish perfusion. Unfortunately, these are all unsuccessful. The patient ultimately requires below the knee amputation.

Vignette 35 Scenario
A 39-year-old female with a history of chronic otitis media undergoes middle ear surgery for cholesteatoma. When evaluated 3 weeks postoperatively she complains of altered taste and numbness of her anterior tongue. Follow up at one year reveals no evidence of cholesteatoma recurrence, but persistence of her taste alteration and sensory disturbance. She was placed on 50 mg of amitriptyline daily with symptom improvement, but without symptom elimination.

© 2025 Hidalgo CM et al. *JAMA Network Open*.

eTable 2. Literature Review Oto—HNS Complication Frequency Summary

Complication	*Frequency*
Wound	
Infection (SSI)	2874
Hemorrhage	317
Flap Complication	1672
Dehiscence	1434
Fistula	2429
Anastomotic leak	47
Hematoma	1112
Necrosis	389
Seroma	185
Sialocele	7
Chyle leak	35
Nasopharynx Stenosis/Stricture	66
Paresthesia	182
Scar Revision/Cosmesis/Cosmetic Defect	93
Donor Site Complication	1026
Miscellaneous Wound Complications	1220
Unspecified Surgical Complications	185
Cardiovascular	
ACS or MI	97
Arrhythmia	54
Cardiac Arrest	115
Heart Failure	9
Hypertension	44
Carotid Blow Out	8
IJV Rupture	1
Cardiovascular Event	6
Respiratory	
Pneumonia	428
Pulmonary Embolism	9
Respiratory Failure	29
Unplanned Tracheostomy	7
ARDS	34
Loss of Airway	1
Aspiration	1
Reintubation	28
Failure to Wean from Ventilator	4
Respiratory Events	68
Neurologic	
TIA	3
Stroke	45
Seizure	1
Altered Mental Status	267
CSF leak	54

© 2025 Hidalgo CM et al. *JAMA Network Open*.

Frey Syndrome	184
Permanent FNP	154
Transient FNP	649
Permanent RLN Palsy	134
Transient RLN Palsy	462
Dysphagia	5
VC Paralysis/Voice Change/Hoarseness	105
Hearing Loss	17
Dizziness	42
CN XI Palsy	84
Other CN Palsies	118
Headache	23
Meningitis	13
Anoxic Brain Injury	1
Alcohol Withdrawal	42
Neurologic Event	45
Hematologic	
DVT	729
Sepsis	53
Anemia	106
Postoperative Transfusion	5
Genitourinary	
Urinary Tract Infection	44
Acute Kidney Injury	24
Renal Failure	2
Genitourinary Complications	25
Gastrointestinal	
Ileus	6
Nausea	1794
Small Bowel Ischemia	1
Abdominal Wound Complications	27
Electrolyte Disturbance	158
Gastrostomy Tube Dependency	16
Other	3
Endocrine	
Hypocalcemia	957
Hypothyroidism	269
Hypoparathyroidism	487
Endocrine Event	56
Unspecified Composite Complications	264
Hospital Course	
Acute Pain	14
ICU Admission	25
Coma	5
Prolonged Hospitalization	99
Readmission	119
Reoperation	615
Death	285

© 2025 Hidalgo CM et al. *JAMA Network Open*.

Overall Complications	21850

SSI, surgical site infection; ACS, acute coronary syndrome; MI, myocardial infarction; IJV, internal jugular vein; ARDS, acute respiratory distress syndrome; TIA, transient ischemic attack; CSF, cerebrospinal fluid; FNP, facial nerve palsy; RLN, recurrent laryngeal nerve; VC, vocal cord; CN, cranial nerve; DVT, deep vein thrombosis; ICU, intensive care unit

© 2025 Hidalgo CM et al. *JAMA Network Open*.

eTable 3. Duration of Harm: Distribution of SME Responses[a]

Clinical Vignette	Complication within Vignette	Number of SMEs who graded Temporary	Number of SMEs who graded Permanent	Number of SMEs who graded Unknown	Authors Suggestion
**7**	Endocrine dysfunction	1	13	0	Permanent
**9**	Airway control	8	0	1	Temporary
**12**	CN II injury	1	3	2	Permanent
**15**	Vascular injury	2	2	1	Temporary
**18**	CVA/Stroke	0	4	6	Permanent
**25***	STEMI	3	0	3	Temporary
**29****	CN X injury	0	0	5	Unknown
**30**	Dysphagia	2	3	1	Temporary
**33**	SSI	4	1	1	Temporary

SME, subject matter expert; CN, cranial nerve; STEMI, ST elevation myocardial infarction; SSI, surgical site infection
**3 SMEs left duration of harm blank*
***1 SME did not put CN X injury as a complication*
[a] *For vignettes with multiple complications, the complication with the longest duration was chosen for this analysis*

© 2025 Hidalgo CM et al. *JAMA Network Open*.
