# Supplementary material for: Modified Clavien-Dindo Classification for Adverse Events in Otolaryngology–Head and Neck Surgery
Source: JAMA Netw Open. 2025 Oct 27;8(10):e2539761. doi: 10.1001/jamanetworkopen.2025.39761 (PMC12559966; doi:10.1001/jamanetworkopen.2025.39761)
Supplement: Supplement 2. — Data Sharing Statement [file jamanetwopen-e2539761-s002.pdf]

Data Sharing Statement

Hidalgo. Modified Clavien-Dindo Classification for Adverse Events in Otolaryngology–Head and Neck Surgery. *JAMA Netw Open*. Published October 27, 2025. doi:10.1001/jamanetworkopen.2025.39761

Data
Data available: No
